# Action of nicotinic acid on the reversion of hypoxic-inflammatory link on 3T3-L1 adipocytes

**DOI:** 10.1186/s12944-016-0260-1

**Published:** 2016-05-10

**Authors:** Renata Nakamichi, Erika Prates Miranda, Sylvia Madeira de Vergueiro Lobo, Vivian Regina Tristão, Maria Aparecida Dalboni, Beata Marie Redublo Quinto, Marcelo Costa Batista

**Affiliations:** Nephrology Division, Department of Medicine, Universidade Federal de São Paulo, Rua Pedro de Toledo 781, Vila Clementino, São Paulo, Brazil; Universidade Nove de Julho, São Paulo, Brazil; Dialysis Unit, Intensive Care Center, Hospital Israelita Albert Einstein, São Paulo, Brazil; Division of Nephrology, Tufts University School of Medicine, Boston, USA

**Keywords:** Hypoxia, Inflammation, Obesity, Adipocyte, Nicotinic acid

## Abstract

**Background:**

Hypoxia resulting from adipocyte expansion is considered the basis of the inflammatory milieu observed in Metabolic Syndrome. Nicotinic acid can act on adipocytes interfering on the inflammatory response. In this study, we investigated the role of HIF-1 α (hypoxia-inducible factor -1 alpha) in the inflammatory process induced by hypoxia. The effect of nicotinic acid on the PPARs (peroxisome proliferator-activated receptors) expression during the inflammatory response was assessed over its action under HIF-1 α in 3T3-L1 adipocytes submitted to hypoxia.

**Methods:**

3T3-L1 adipocytes were pre-treated with nicotinic acid and incubated under hypoxic conditions. The level of adipokines and HIF-1 α were quantified using immunoassays. Adipokine expression was measured using real-time PCR, whereas PPARs and HIF-1 α expression were analyzed by western blot. The statistical significance of the differences between variables studied was determined by analysis of variance (ANOVA) complemented by Bonferroni’s test.

**Results:**

The results demonstrated an increase in leptin and PAI-1 (plasminogen activator inhibitor-1) expression, while adiponectin production decreased under hypoxia. In parallel, induction with hypoxia enhanced HIF-1 α expression, despite causing reduced expression of PPAR α and PPAR γ. However, nicotinic acid reversed adipokine modulation under hypoxic conditions, leading to decreased HIF-1 α expression and increased PPARs expression.

**Conclusions:**

Our findings suggest that nicotinic acid blunt the inflammatory response resulting from hypoxia by the reduction of HIF-1 α expression and concomitant increase of PPARs α and γ expression in 3T3-L1 adipocytes.

## Background

Obesity, a world epidemic disease, is characterized by the expansion of adipose tissue mass, resulting in a state of chronic low-grade inflammation, which contributes to the development of cardiovascular diseases [1]. Oxygen deprivation, a result of such tissue expansion, is the component leading to an increase of inflammatory proteins in the obesity pathogenesis [2]. Low oxygen tension induces adaptive response in cells to conserve oxygen for vital metabolic functions, these changes occur through the activation of a specific transcription factor called hypoxia-inducible factor 1 (HIF-1). HIF-1 is a heterodimer composed of HIF-1 α, which is considered the molecular oxygen sensor, and HIF-1 β, which is associated with the receptor nuclear translocator [3]. Wang et al. reported that low oxygen tension in human adipocytes induced a marked increase in HIF-1 α expression with a concomitant increase in the secretion and expression of several adipokines, including leptin and plasminogen activator inhibitor-1 (PAI-1), whereas adiponectin was inhibited during such oxygen deprivation [4]. Indeed, subsequent studies demonstrated that, *in vitro*, hypoxia downregulates the expression of peroxisome proliferator-activated receptors ɑ and γ (PPAR α and PPAR γ) [5, 6]. These findings may provide evidence of an adaptive response to inflammatory processes during a hypoxic state.

Nicotinic acid targets adipocytes through the activation of a G-protein-coupled receptor (GPR-109 A) which is highly expressed in adipose tissue [7]. The activation of GPR-109 A results in an increased adiponectin levels and an attenuation in leptin and PAI-1 secretion from adipocytes [8]. Digby et al. found that nicotinic acid suppressed pro-inflammatory proteins and upregulated the production of atheroprotective adiponectin in 3T3-L1 adipocytes [9]. However, the mechanism by which nicotinic acid affects the expression of PPAR γ and PPAR ɑ is not fully understood. Yang et al. also reported a significant increase of PPAR γ mRNA expression in hypercholesterolemic rabbit adipocytes treated with the drug, suggesting that this receptor can also stimulate the transcription of genes involving in the regulation of several inflammatory adipokines [10].

In the present study, we investigated the link between inflammatory response induced by oxygen deprivation and the role of PPAR γ and PPAR α under hypoxic conditions. We speculate whether the potential benefits, observed from nicotinic acid in mitigation of the inflammatory response associated with hypoxia, are mediated by HIF-1 α under PPAR α and PPAR γ activation.

## Methods

### Cell culture

Murine fibroblasts of the immortalized 3T3-L1 cell line in the 7^th^ passage were obtained from the American Type Culture Collection and were grown in DMEM-HG (Dulbecco’s modified Eagle’s medium high glucose, Gibco, NY, USA) containing 10 % (v/v) fetal bovine serum (FBS, Gibco, NY,USA). Confluent cells were induced to differentiate into adipocytes, according to a previously established protocol [11, 12].

### Cell treatment

**Fig. 1:**
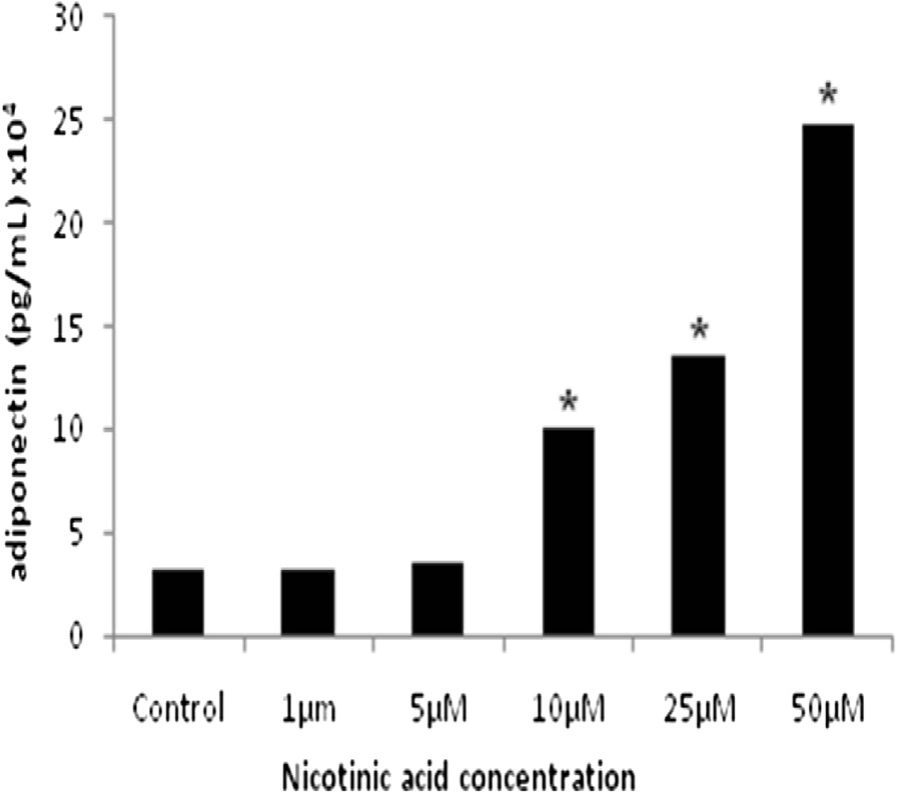
Dose effects of nicotinic acid on adiponectin secretion. Serial dilutions of nicotinic acid on adiponectin secretion in 3T3-L1 adipocytes; *10 μM, 25 μM, 50 μM groups versus Control group, *p* < 0.05. The statistical significance of the differences between variables studied was determined by analysis of variance (ANOVA) - *n* = 6

On the 10^th^ day following the beginning of differentiation, the adipocytes were pre-treated with nicotinic acid (10 μmol/L) (Sigma Aldrich, St. Louis, MO, USA) or the vehicle control (non-supplemented DMEM HG medium) for 24 h at 37 ° C in an incubator containing 5 % CO_2_ and ideal humidity conditions. After the nicotinic acid treatment, the cells were exposed to hypoxia (1 % O_2_, 94 % N_2_ and 5 % CO_2_), sealed and placed at 37 ° C for different periods of time (4, 8 and 12 h). Serial dilutions of nicotinic acid had been previously tested to obtain a working concentration of 10 μmol/L (Fig. 1). Oxygen saturation was performed in all groups using a blood gas analyzer (ABL5 - *Radiometer Copenhagen*, Holliston, MA, USA) from laboratory of *Hospital do Rim* (data not shown).

### Differentiation of fibroblasts into adipocytes by oil red staining

The differentiation of 3T3-L1fibroblast into adipocytes was evaluated every 24 h from the 10^th^ day following the beginning of differentiation by observing the morphologic appearance of the cells with a light microscope. On the 10^th^ day, the formation of lipid droplets, a morphologic appearance characteristic of adipocytes, was confirmed by oil red (Sigma Aldrich, St. Louis, MO, USA) staining. (data not shown).

Initially, 60 mg of oil red stain were dissolved in 20 mL of isopropanol. After 300 μL of this solution were diluted in water in 6:4 proportions. Culture medium was aspirated and the wells were washed twice with phosphate-buffered saline (PBS). Next, 4 % paraformaldehyde (diluted in PBS) was added in a volume sufficient to cover the cells. After 30 min at room temperature, the medium was aspirated and the wells were washed 3 times with PBS. The cells were then incubated in oil red solution (300 μL) for 2 h, at 37 ° C. Afterward, the medium was aspirated, and the plates were washed thrice with distilled water and placed into the incubator, at 37 ° C to dry. After this step, the cells were examined with optic microscope and photographs were taken.

### Determination of cell viability

We could observe by trypan blue (Sigma Aldrich, St. Louis, MO, USA) exclusion that from the 4^th^ to the 10^th^ day, at the beginning of differentiation, cell viability was about 95 % (data not shown). The cells were trypsinized and suspended in 10 mL of PBS. A 25 μL aliquot of this cell suspension was mixed with 75 μL of trypan blue stain. From this mixture, 10 μL were placed in a Neubauer chamber to count viable nucleus cells stained in red.

### Measurement of HIF-1 α and adipokines by ELISA

Total HIF-1 α in the cell lysates was quantified using an immunoassay kit (ELISA) (R&D Systems, Minneapolis, MN, USA), according to the manufacturer’s protocol. The cells were lysed as previously described by Wang et al. [4]. The secretion of the adipokines in 3T3-L1 adipocytes was determined by measuring the leptin, PAI-1 and adiponectin concentrations in the cell culture medium using a commercial ELISA kit (Linco Research – Millipore, St. Charles, MO, USA; Wuhan EIAAB Science CO., LTD, Wuhan, China and R&D System, Minneapolis, MN, USA, respectively), according to the manufacturer’s protocol.

### mRNA extraction and cDNA synthesis

Total mRNA from mature adipocytes was extracted with QuantiTeck kit (Qiagen, Hilden, NRW, German) according to the manufacture’s protocol. After, cDNA was prepared using 1 μg of total RNA by the appropriate kit (RevertAid H Minus First Strand cDNA Synthesis Kit, Thermo Scientific, Pittsburgh, PA, EUA).

### Analysis of adipokines expression by real time PCR

An aliquot of cDNA was mixed with the specific primers for the genes of interest, along with deoxynucleotides, Taq polymerase and the fluorescent stain SybrGreen (Quantitect kit, Qiagen, Hilden, NRW, German). Primers for the genes of interest were designed using the Primer Express® program (Applied Biosystems, Gibco, NY, USA) and were based on the gene sequences obtained from GenBank. The primer sequences are described in the Table 1.

**Table 1:** Primer sequence for Real -Time PCR

Gene	Forward sequence Reverse sequence	Reference
Adiponectin	5’- ACAGCCTTTGTCATCTCAGC-3’ 5’-CCGAACCACAAAGAGAAAGGA-3’	XR_376389
Leptin	5-’CATCTGCTGGCCTTCTCCAA-3’ 5'-ATCCAGGCTCTCTGGCTTCTG-3’	NM_008493.3
PAI-1	5’-ACAGCCTTTGTCATCTCAGCC-3’ 5’-CCGAACCACAAAGAGAAAGGA-3’	NM_013556.2
HPRT	5-’CTCATGGACTGATTATGGACAG-3’ 5’-GCAGGTCAGCAAAGAACTTATA-3’	NM_013556.2

Reactions utilizing the specific primers were systematically performed. The specificity of the product was confirmed by melting point curve analysis, as well as by conventional agarose gel electrophoresis. The products were quantified and compared to controls based on the analysis of the number cycles necessary to obtain a given fluorescence value in the log-linear phase of the reaction. Reactions utilizing the primer for the housekeeping gene HPRT (hypoxanthine phosphoribosyl transferase) were systematically performed, and all values obtained for the genes of interest were normalized with HPRT expression.

### Analysis of HIF-1 ɑ, PPAR α and PPAR γ by western blot

Total protein was extracted from 3T3-L1 adipocytes. Next, the samples were concentrated using Centrifugal Filter Devices (Milipore, St. Charles, MO, USA) and a Speed Vac Concentrator (SPD111V, Thermo Scientific, Pittsburgh PA, USA). Total protein was quantified using the BCA Protein Assay reagent (Thermo Scientific, Pittsburgh PA, USA), and 20 μg protein was separated by SDS-PAGE and transferred onto nitrocellulose membranes. These membranes were blocked and probed with mouse monoclonal anti-HIF-1 ɑ (Milipore, St. Charles, MO, USA) or rabbit polyclonal anti-PPARγ (Milipore, St. Charles, MO, USA) or mouse monoclonal anti-PPAR ɑ (Milipore, St. Charles, MO, USA) or mouse anti-actin (Sigma Aldrich, St. Louis, MO, USA) as the primary antibody. The membranes were then submitted to peroxidase-conjugated goat anti-mouse IgG (Milipore, St. Charles, MO, USA) or peroxidase-conjugated goat ant-rabbit IgG (Milipore, St. Charles, MO, USA).

The specific proteins were observed following incubation with the chemiluminescent HRP substrate (Immobilon Western, Millipore, St. Charles, MO, USA). The densitometric analyses of the proteins of interest were performed, using UVI band software (Univitec – Cambridge, England).

### Statiscal analysis

Statistical analysis was performed using the SPSS 20.0 software. The results for each individual group were expressed as mean ± standard deviation. The statistical significance of the differences between variables studied was determined by analysis of variance (ANOVA) complemented by Bonferroni’s test. The level of statistical significance was 0.05 for all tests.

## Results

### Total HIF- 1 α intracellular concentration and expression and PPAR α and PPAR γ expression

**Fig. 2:**
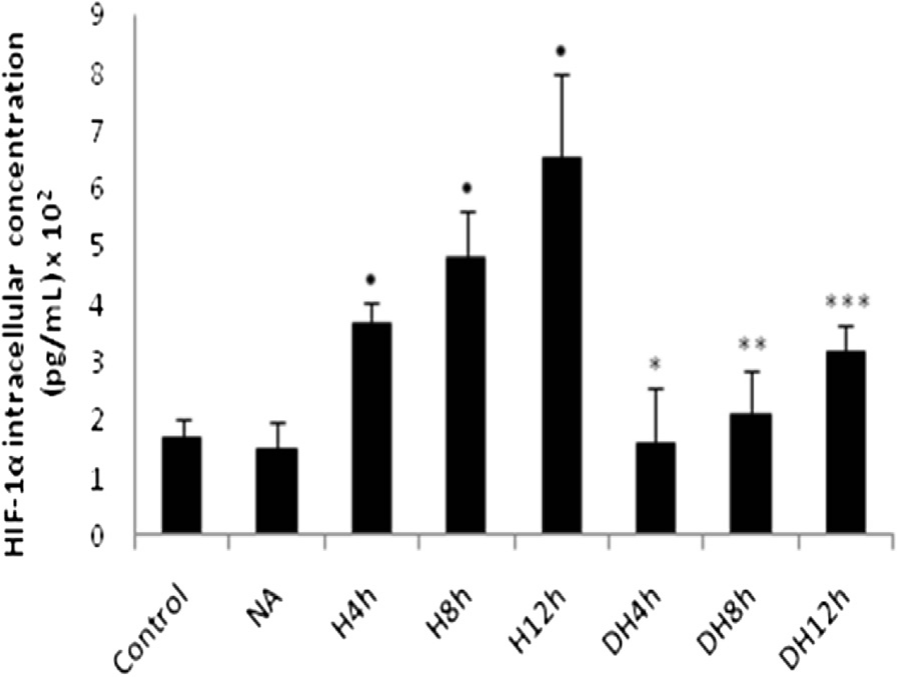
HIF-1 ɑ intracellular concentration in adipocytes pre-treated with nicotinic acid and submitted to hypoxia. HIF-1α intracellular concentration in 3T3-L1 adipocytes; ^•^H4 h, H8 h, H12 h groups versus Control group; * DH4 h versus H 4 h; ** DH 8 h versus H8 h; *** DH 12 h versus H12 h, *p* < 0.05. The statistical significance of the differences between variables studied was determined by analysis of variance (ANOVA) - *n* = 10; NA: Nicotinic acid, H: hypoxia; DH: Drug + Hypoxia

**Fig. 3:**
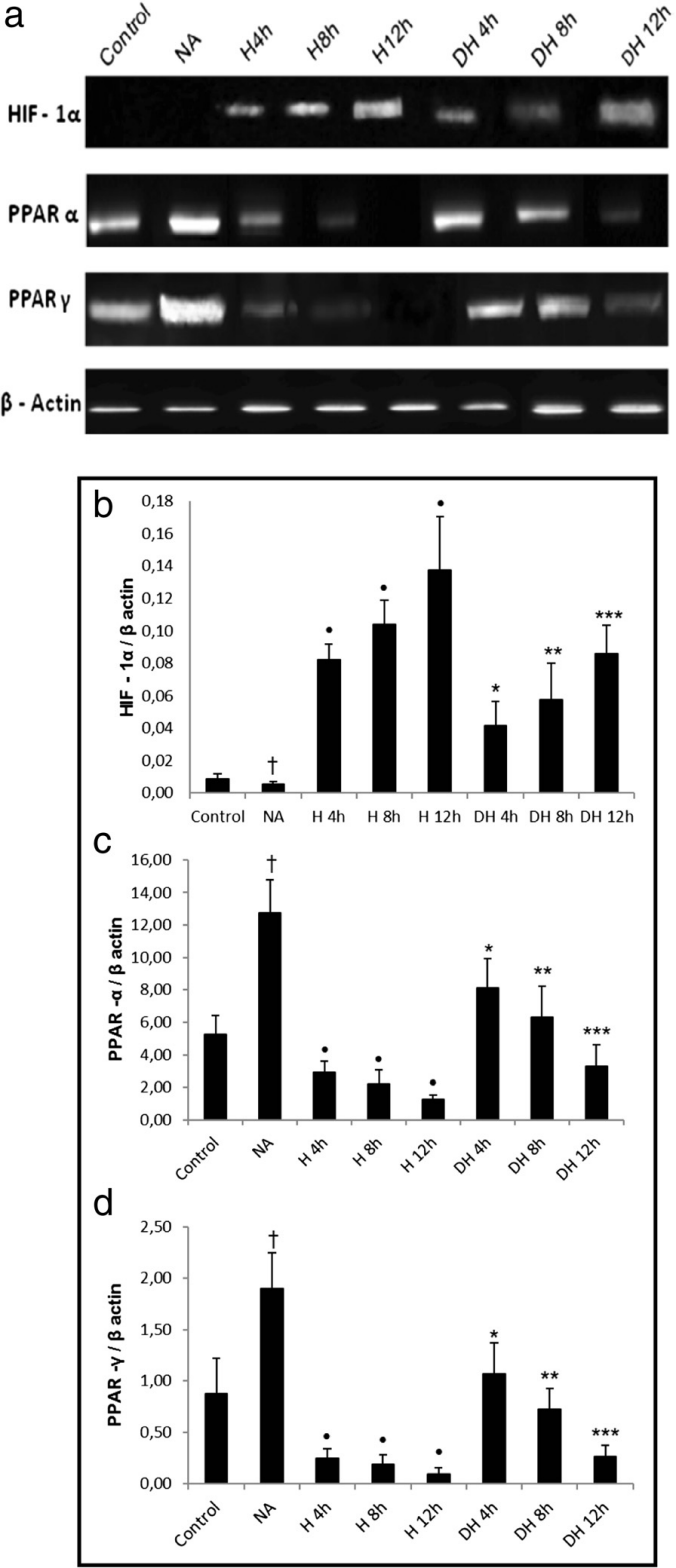
Expression of HIF-1 ɑ, PPAR ɑ and PPAR γ in adipocytes pre-treated with nicotinic acid and submitted to hypoxia. **a** Expression of HIF-1 α, PPAR α and PPAR γ in 3T3-L1 adipocytes through Western blot. **b** Densitometric analysis of HIF-1 α by Western blot. **c** Densitometric analysis of PPAR α expression by Western Blot. **d** Densitometric analysis of PPAR γ by Western blot. ^†^NA versus Control group; ^•^H 4 h, H 8 h, H 12 h groups versus Control group; *DH 4 h versus H 4 h; **DH 8 h versus H 8 h; ***DH 12 h versus H 12 h, *p* < 0.05. The statistical significance of the differences between variables studied was determined by analysis of variance (ANOVA) - *n* = 6; NA: Nicotinic acid; H: Hypoxia; DH: Drug + Hypoxia

We observed a gradual increase in HIF-1 ɑ intracellular concentration during the different periods of hypoxia compared to the baseline conditions (Fig. 2). However, we verified that the pre-treatment with nicotinic acid decreased the total HIF-1 α intracellular concentration in adipocytes incubated under hypoxic conditions for different periods of time compared to cells cultured only under the respective periods of oxygen deprivation. The treatment with nicotinic acid didn’t exert effects on HIF-1 α compared to baseline conditions (Fig. 2). Analysis of the western blot images demonstrated an enhanced HIF-1 α expression and showed a decrease in PPAR α and PPAR γ expression in adipocytes exposed to different periods of hypoxia compared to the baseline conditions in 3T3-L1 adipocytes (Fig. 3). Moreover, we observed that adipocytes pre-treated with the drug had also decreased HIF-1 α expression and enhanced PPAR α and PPAR γ expression during the different periods of hypoxia compared to the cells submitted to only oxygen deprivation (Fig. 3).

### Leptin and PAI-1 secretion and expression

**Fig. 4:**
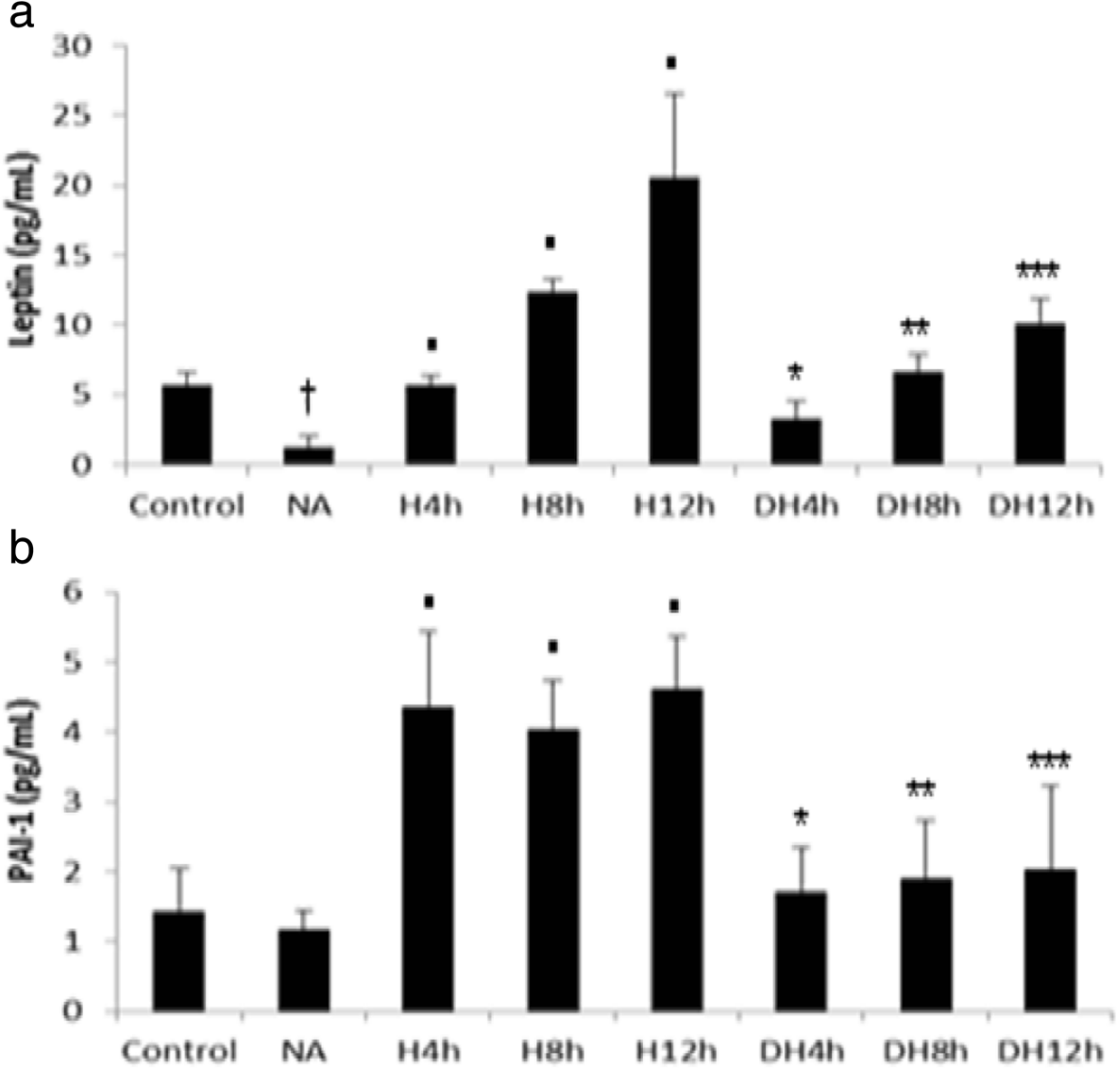
Secretion of leptin and PAI-1 in adipocytes pre-treated with nicotinic acid and submitted to hypoxia. **a** Secretion of leptin in 3T3-L1 adipocytes. **b** Secretion of PAI-1 in 3T3-L1 adipocytes; ^†^NA versus Control group; ^•^H 4 h, H 8 h, H 12 h groups versus Control group; *DH 4 h versus H 4 h; **DH 8 h versus H 8 h; ***DH 12 h versus H 12 h, *p* < 0.05. The statistical significance of the differences between variables studied was determined by analysis of variance (ANOVA) - *n* = 10; NA: Nicotinic acid; H: Hypoxia; DH: Drug + Hypoxia

Hypoxia activated the secretion of leptin in the different periods compared to the control group (Fig. 4a). We also verified an increase in PAI-1 secretion in adipocytes submitted to the different periods of hypoxia compared to the control group (Fig. 4b). The pre-treatment with nicotinic acid suppressed leptin secretion in adipocytes exposed to the different periods of oxygen deprivation compared to cells cultured only under the respective periods of hypoxia (Fig. 4a). Furthermore, we observed a decrease in PAI-1 secretion in adipocytes pre-treated with nicotinic acid and submitted to hypoxia compared to the respective periods of oxygen deprivation (Fig. 4b). The treatment with nicotinic acid decreased the leptin secretion compared to control group (Fig. 4a), but didn’t exert effects on PAI-1 secretion (Fig. 4b). The analysis of real time PCR demonstrated an increase of leptin and PAI-1 expression in adipocytes submitted to hypoxia compared to control group (Table 2) and a suppression of these adipokines expression in adipocytes pre-treated with nicotinic acid and exposed to hypoxia compared to cells only submitted to the respective periods of oxygen deprivation. Moreover, the treatment with nicotinic acid decreased leptin and PAI-1 expression (Table 2).

**Table 2:** Expression of leptin, PAI-1 and adiponectin in 3T3-L1 adipocytes

Relative mRNA expression	Control	NA	H 4 h	H 8 h	H 12 h	DH 4 h	DH 8 h	DH 12 h
Leptin	0.2 ± 0.3	0.4 ± 0.007^a^	136.4 ± 17.0^b^	320.7 ± 72.3^b^	571.7 ± 53.6^b^	5.06 ± 3.1^c^	26.8 ± 9.4^d^	74.7 ± 14.1^e^
PAI-1	1.4 ± 0.4	0.04 ± 0.04^a^	7.3 ± 2.7^b^	16.0 ± 2.7^b^	30.6 ± 7.4^b^	0.2 ± 0.3^c^	0.6 ± 0.4^d^	8.0 ± 3.5^e^
Adiponectin	255.1 ± 130.7	1195.7 ± 418.7^a^	2.9 ± 2.0^b^	0.6 ± 0.2^b^	0.08 ± 0.02^b^	278.7 ± 146.7^c^	214.3 ± 62.7^d^	22.2 ± 9.8^e^

Expression of adipokines in adipocytes: ^a^NA versus Control group; ^b^H 4 h, H 8 h, H 12 h groups versus Control group; ^c^DH 4 h versus H 4 h; ^d^DH 8 h versus H 8 h; ^e^DH 12 h versus H 12 h, *p* < 0.05. The statistical significance of the differences between variables studied was determined by analysis of variance (ANOVA) - n=10; NA: Nicotinic acid; H: Hypoxia; DH: Drug + Hypoxia

### Adiponectin secretion and expression

**Fig. 5:**
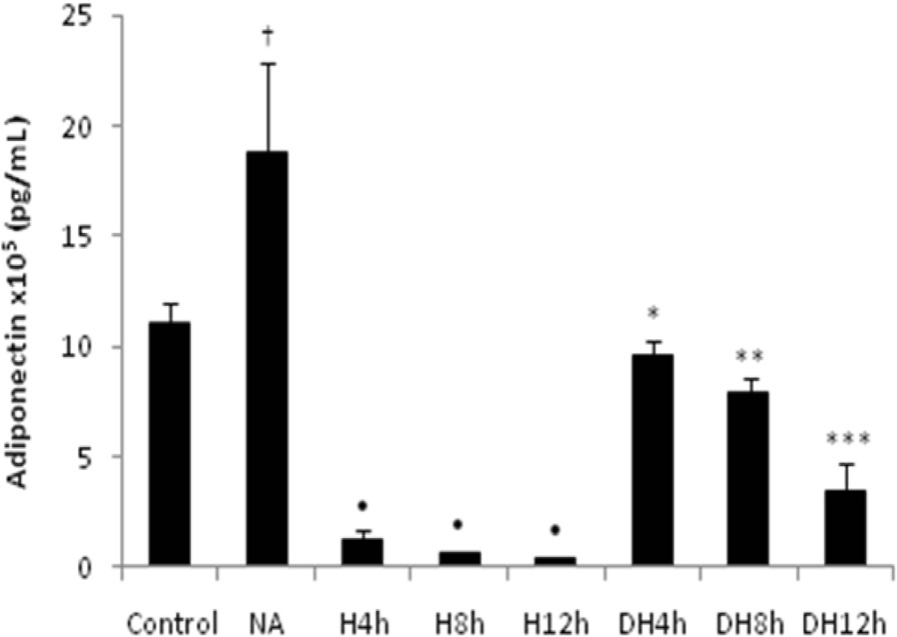
Secretion of adiponectin in adipocytes pre-treated with nicotinic acid and submitted to hypoxia. Secretion of adiponectin in 3T3-L1 adipocytes; ^†^ NA versus Control group; ^•^ H4 h, H8 h, H12 h groups versus Control group; * DH 4 h versus H 4 h; ** DH 8 h versus H 8 h; *** DH 12 h versus H 12 h, *p* < 0.05. The statistical significance of the differences between variables studied was determined by analysis of variance (ANOVA) - *n* = 10; NA: Nicotinic acid; H: Hypoxia; DH: Drug + Hypoxia

Hypoxia decreased the expression (Table 2) and secretion of adiponectin compared to control group (Fig. 5). Pre-treatment with nicotinic acid increased the expression (Table 2) and secretion of adiponectin in the cells submitted to the different periods of oxygen deprivation compared to the adipocytes only exposed to the respective periods of hypoxia (Fig. 5). The treatment with nicotinic acid enhanced the expression (Table 2) and the secretion of adiponectin compared to control group (Fig. 5).

### Correlation between HIF- 1α and the leptin, PAI-1and adiponectin secretion

**Fig. 6:**
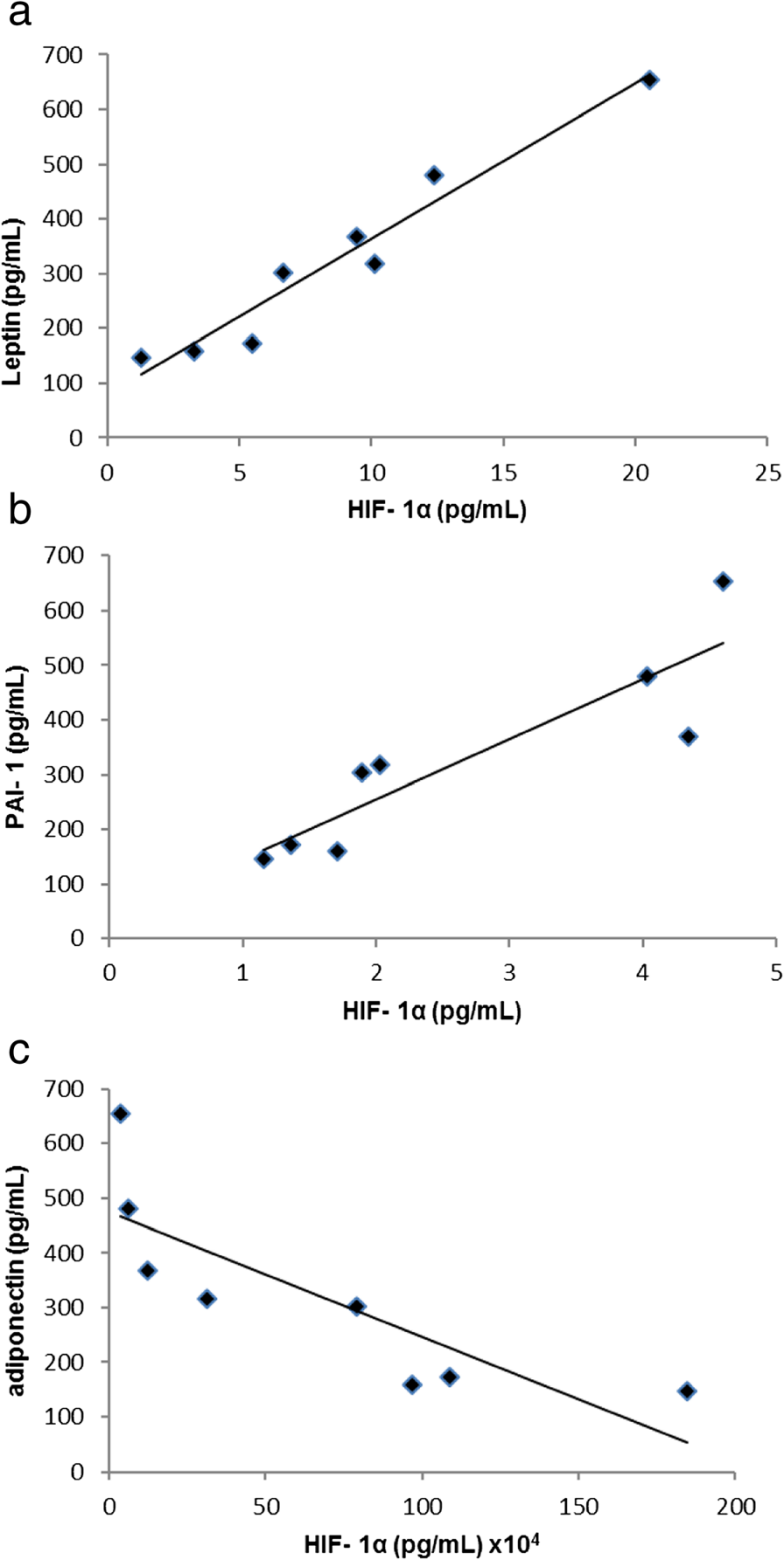
Correlation between HIF-1 α concentration and adipokines secretion. **a** Correlation between HIF-1 α and leptin, *R* ^2^ = 0.94, *p* = 0.01. **b** Correlation between HIF-1 α and PAI-1, *R* ^2^ = 0.80, *p* = 0.01. **c** Correlation between HIF-1 α and adiponectin, *R* ^2^ = 0.70, *p* = 0.01. The statistical significance between variables studied was determined by Pearson Correlation- *n* = 10

We observed a correlation between HIF- 1alpha and leptin (*R*^2^ = 0.95) (Fig. 6a), PAI-1(*R*^2^ = 0.79) (Fig. 6b) and adiponectin secretion (*R*^2^ = 0.68) (Fig. 6c).

## Discussion

There is increasing evidence that low oxygen tension impairs adipocyte function, modulates the expression of adipokines and contributes to metabolic disorders through the induction of adaptive responses. In the present study, we found that hypoxia mediates an increase in inflammatory proteins, such as leptin and PAI-1, while resulting in a decrease in adiponectin expression and secretion. In parallel, we demonstrated an enhancer of HIF-1 ɑ in hypoxic 3T3-L1 adipocytes. In fact, Hosogai et al. showed that HIF-1 α downregulated the expression of adiponectin mRNA and amplified the synthesis of PAI-1 in the adipose tissue of obese mice [2]. Halberg et al. reported that transgenic expression of a constitutively active form of this transcription factor induced glucose tolerance in rats [13]. These results corroborate the hypothesis that HIF-1 α plays an important role in the transmission of the hypoxic response in adipose tissue. HIF-1 α is considered to be the major molecular oxygen sensor that regulates the expression of a large group of genes related to several physiological processes such as erythropoiesis, angiogenesis and glycolysis [14].

The precise role of HIF-1 α in modulating adipokine production remains poorly characterized, but recent studies have indicated that PPARs are involved in this mechanism. Our findings demonstrated a reduction in PPAR α and PPAR γ expression with a concomitant increase in HIF-1 ɑ expression; at the same time, we observed changes in the synthesis and release of adipokines under conditions of oxygen deprivation. Pino et al. confirmed the role of PPAR γ in regulating the response of white adipose tissue to hypoxia [5]. On the basis of our findings, it is possible that HIF-1 α modulates leptin, PAI-1 and adiponectin secretion and expression through the PPARs pathway. Although this mechanism is not fully understood, a number of studies have emphasized such a possibility. Narravula and Colgan associated the downregulation of PPAR α with HIF-1 α induction in intestinal epithelial cells. In parallel, they identified a binding site for HIF-1 α on the antisense strand of the PPAR α gene, resulting in an increase in inflammation-related gene products [6]. Additionally, Nicolla et al. observed that HIF-1 α requires cofactors, such as PPAR γ, to facilitate the expression of hypoxia-associated genes by interfacing with components bound to the regulatory elements in the corresponding promoter regions under low oxygen tension in rats [15]. The expansion of adipose tissue results in the infiltration of macrophages and the production of inflammatory proteins, which reduces PPAR γ activity on select target genes and results in an increase in HIF-1 α activity. This increased activity leads to the suppression of beneficial adipokine expression, such as adiponectin, and the increased expression of leptin and PAI-1 [16].

We also studied the effect of nicotinic acid on the attenuation of the inflammatory response associated with hypoxia. In this regard, we showed a modulation of adipokines in hypoxic adipocytes exposed to nicotinic acid. Recent studies have demonstrated the action of nicotinic acid on the attenuation of inflammatory process through the activation of its specific receptor GPR-109 A [7], highly expressed in adipose tissue [9, 10]. Indeed, Plaisance et al. showed the role of GPR-109 A in the regulation of adiponectin production, demonstrating an increase in the adiponectin secretion through the activation of the receptor in 3T3-L1 adipocytes [17].

As previously described, the importance of HIF- 1 α in the adaptation of cells to low oxygen tension and its effects on the expression of genes involving in angiogenesis, glucose metabolism and erythropoiesis make this transcription factor an attractive target [3]. In fact, several studies have demonstrated that HIF-1 α is implicated in metabolic disorders in other tissues as liver, lung, breast [18, 19]. Additionally, natural antagonists or antisense strategies have been applied in mouse models, and therapeutic strategies have focused on developing new small-molecule HIF-1 ɑ inhibitors [20]. Recently, Jiang et al. demonstrated that acriflavine (ACF), a HIF-1 inhibitor, increased the expression of adiponectin, suggesting its potential as a therapeutic treatment for obesity and type 2 diabetes [21]. Moreover, Boovanahalli et al. suggested that isonicotinic and nicotinic acid derivates may inhibit HIF-1 α activity [22].

Less is known about whether HIF-1 α can be blocked by its inhibitors or whether nicotinic acid can reverse the inflammation mediated by this transcription factor. On the basis of our findings, nicotinic acid acts by reducing the expression of inflammatory adipokines and increasing the expression of adiponectin, which is related to the enhancement of PPAR γ and PPAR α expression observed in 3T3-L1 adipocytes. Therefore, we speculate that nicotinic acid inhibits HIF-1 α through the PPARs pathway, thus resulting in the reversal of the inflammatory response mediated by hypoxia. Increasing amounts of evidence suggest that PPAR γ also has high affinity for certain drugs, such as nicotinic acid [23]. It has been shown that nicotinic acid induces PPAR γ expression and the transcriptional activation in macrophages via GPR-109 A mediated induction of prostaglandin synthesis pathways [24]. Rubic et al. suggested that nicotinic acid acts on the translocation and transcription of PPAR γ, which indirectly stimulates prostaglandin D2 and consequently prostaglandin J2, an endogenous ligand of PPAR γ, thereby increasing adiponectin expression [25]. Indeed, this mechanism was also observed to be the responsible mechanism, since leptin production decreased after treatment with acipimox, a nicotinic acid analogue [26].

## Conclusion

In conclusion, the present study demonstrated that hypoxia induces adipokine modulation, while increasing HIF-1 ɑ secretion and expression. This was in parallel to its suppression of PPAR ɑ and PPAR γ under hypoxic conditions, suggesting a possible link between the two pathways. Our findings also propose, that nicotinic acid is related to the amelioration of hypoxic effects in 3T3-L1 adipocytes. Whether the results obtained can be translated to the clinical setting is still a matter of debate and should be the objective of future studies.

## Abbreviations

ACF: acriflavine
DMEM-HG: Dulbecco’s modified Eagle’s medium high glucose
FBS: fetal bovine serum
GPR: G-protein-coupled receptor
HIF-1: hypoxia-inducible factor- 1
HPRT: hypoxanthine phosphoribosyl transferase
PAI-1: plasminogen activator inhibitor-1
PPAR: peroxisome proliferator-activated receptors
